# Integrated Transcriptomic and Metabolomic Analysis Reveals Tissue-Specific Flavonoid Biosynthesis and MYB-Mediated Regulation of UGT71A1 in *Panax quinquefolius*

**DOI:** 10.3390/ijms26062669

**Published:** 2025-03-16

**Authors:** Yumeng Wang, Jiaxin Zhang, Ping Wang, Yongkang Li, Yihan Wang, Yan Yan, Junwen Chi, Jiankang Chen, Junmei Lian, Xiangmin Piao, Xiujuan Lei, Ying Xiao, Jeremy Murray, Micheal K. Deyholos, Yingping Wang, Peng Di, Jian Zhang

**Affiliations:** 1 State Local Joint Engineering Research Center of Ginseng Breeding and Application, College of Chinese Medicinal Materials, Jilin Agricultural University, Changchun 130118, Chinaping@mails.jlau.edu.cn (P.W.); 20231693@mails.jlau.edu.cn (J.C.); chenjk@mails.jlau.edu.cn (J.C.); lian@mails.jlau.edu.cn (J.L.);; 2 The MOE Key Laboratory for Standardization of Chinese Medicines and the SHTCM Key Laboratory for New Resources and Quality Evaluation of Chinese Medicines, Institute of Chinese Materia Medica, Shanghai University of Traditional Chinese Medicine, Shanghai 200032, China; 3 CAS Center for Excellence in Molecular Plant Sciences, 300 Fenglin Road, Shanghai 200032, China; 4 Department of Biology, University of British Columbia, Okanagan, Kelowna, BC V1V 1V7, Canada; 5 Faculty of Agronomy, Jilin Agricultural University, Changchun 130118, China

**Keywords:** flavonoid biosynthesis, American ginseng, *Panax quinquefolius*, transcriptomic and metabolomic integration study

## Abstract

*Panax quinquefolius* is a globally valued medicinal plant rich in bioactive flavonoids, yet the molecular mechanisms underlying their biosynthesis remain poorly understood. In this study, we integrated transcriptomic and metabolomic analyses to investigate tissue-specific flavonoid accumulation and regulatory networks in roots, leaves, and flowers. Metabolomic profiling identified 141 flavonoid metabolites, with flavones, flavonols, and C-glycosylflavones predominantly enriched in aerial tissues (leaves and flowers), while specific glycosides like tricin 7-*O*-acetylglucoside showed root-specific accumulation. Transcriptome sequencing revealed 15,551–18,946 DEGs across tissues, and the reliability of the transcriptomic data was validated by qRT-PCR. KEGG and GO annotation analyses suggested that these DEGs may play a crucial role in the biosynthesis and metabolism of secondary metabolites. From the DEGs, UGTs and MYB TFs were identified and subjected to correlation analysis. Functional validation through in vitro enzymatic assays confirmed that *PqUGT71A1* catalyzes apigenin and naringenin glycosylation at the 7-OH position. Additionally, subcellular localization and yeast one-hybrid assays demonstrated that *PqMYB7* and *PqMYB13* interact with the *PqUGT71A1* promoter and activate its expression.. This study unveils the spatial dynamics of flavonoid metabolism in *P. quinquefolius* and establishes a MYB-UGT regulatory axis, providing critical insights for metabolic engineering and bioactive compound optimization in medicinal plants.

## 1. Introduction

*Panax quinquefolius (P.quinquefolius)*, a species in the Araliaceae family, is one of the world’s most popular medicinal herbs, known for its diverse pharmacological activities [1]. Various tissues of *P. quinquefolius*, including roots, stems, leaves, and flowers, hold significant medicinal and economic value, being widely utilized in pharmaceutical formulations, functional foods, and cosmetic products. *P. quinquefolius* has been traditionally used in herbal medicine for centuries and is now commonly incorporated into dietary supplements, functional foods, and cosmetics. Pharmacological studies have demonstrated its potential therapeutic applications in cardiovascular diseases [2], immune enhancement [3], cancer cell inhibition [4], and cognitive function improvement [5]. Extensive phytochemical investigations have revealed *P*. *quinquefolius* as a rich source of bioactive constituents [6,7], notably triterpenoid saponins (e.g., ginsenosides), flavonoid glycosides, essential minerals (zinc, iron), and phenolic acids.

Flavonoids, a major class of polyphenolic compounds, function as signaling molecules, intermediates, and secondary metabolites in plants [8]. They are among the most biologically active polyphenolic compounds, including flavones, isoflavones, flavonols, isoflavonols, flavanones, isoflavanones, and chalcones [9], and they are widely distributed across the plant kingdom. To date, over 8000 flavonoid compounds [10] have been identified in plants. They are important not only in UV radiation [11] and inhibiting auxin transport [12] it is also important in mitigation of various biotic [13], abiotic stress [14]. Although the biosynthetic pathways of flavonoids have been studied in model and medicinal plants, such as *Arabidopsis thaliana* [15], *Ziziphus jujuba* [16], *Ginkgo biloba* [17], and *Phalaenopsis amabilis* [18], the flavonoid biosynthesis pathways in *P. quinquefolius* remain unclear.

Transcription factors play a crucial role in regulating flavonoid biosynthesis by modulating the expression of key enzyme genes, thereby influencing metabolic pathway activity. Transcriptomics is a powerful tool for investigating functional genes [19], offering valuable insights into differential gene expression and regulatory networks through transcriptome sequencing [20,21]. Metabolomics, a relatively newer “omics” discipline emerging alongside genomics, transcriptomics, and proteomics, provides a direct reflection of biological responses and serves as a key foundation for understanding phenotypic traits [22,23]. It focuses on identifying the types, quantities, and dynamic fluctuations of metabolites within an organism in response to perturbations [24]. Due to the intricate regulatory mechanisms governing secondary metabolite biosynthesis, data from a single omics approach often provide an incomplete picture of the underlying pathways. Multi-omics integration, however, facilitates a more comprehensive exploration of potential regulatory networks within organisms, thereby providing deeper insights into biological mechanisms [25,26]. For instance, an approach that combined transcriptomic and metabolomic analyses was used to reveal the complex metabolic genes involved in the biosynthesis of diterpenoids in different organs of the *Anoectochilus Roxburghii* [27], transcriptomic and metabolomic analyses have been used to reveal the relationship between metabolic processes and peel color in melons [28] and to elucidate flavonoid biosynthesis pathways in *Areca Catechu* [29].

Glycosylation plays a crucial role in the biosynthesis of secondary metabolites by enhancing their water solubility, chemical stability, and influencing their bioactivity and intracellular distribution. UGTs are key enzymes mediating this process, catalyzing the transfer of sugar moieties from donor molecules to acceptor compounds (e.g., flavonoids), thereby modulating their functional properties. In *P. quinquefolius*, flavonoids primarily exist in glycosylated forms, which enhances their solubility and stability within the plant [30]. Flavonoid glycosyltransferases responsible for these modifications remain unidentified in *P. quinquefolius*. In flavonoids, glycosylation typically occurs at hydroxyl groups at positions 3, 5, 7, 3′, 4′, and 5′ [31]. Specific glycosyltransferases catalyze the formation of distinct flavonoid monoglycosides. For example, the pomegranate glycosyltransferase *PgUGT95B2* specifically glycosylates tricetin, a flavone with a trihydroxylated B-ring, at the 4′-OH position [32]. Similarly, *UGT73AC11*, identified in *Arabidopsis*, transfers glucose or xylose to the 7-OH or 4′-OH positions of flavonoids, leading to the formation of glycosides [33].

In this study, transcriptomic and metabolomic analyses were performed on *P. quinquefolius* roots, leaves, and flowers to elucidate flavonoid biosynthetic pathways. Additionally, key TFs involved in metabolite biosynthesis regulation were identified. Furthermore, specific members of the *PqMYBs* and *PqUGTs* families were identified as regulators of flavonoid biosynthesis in *P. quinquefolius*. These findings provide a foundation for further exploration of flavonoid biosynthetic mechanisms and metabolic regulation.

## 2. Results

### 2.1. Tissue-Specific Differential Accumulation of Flavonoid Metabolites in P. quinquefolius

**Figure 1:**
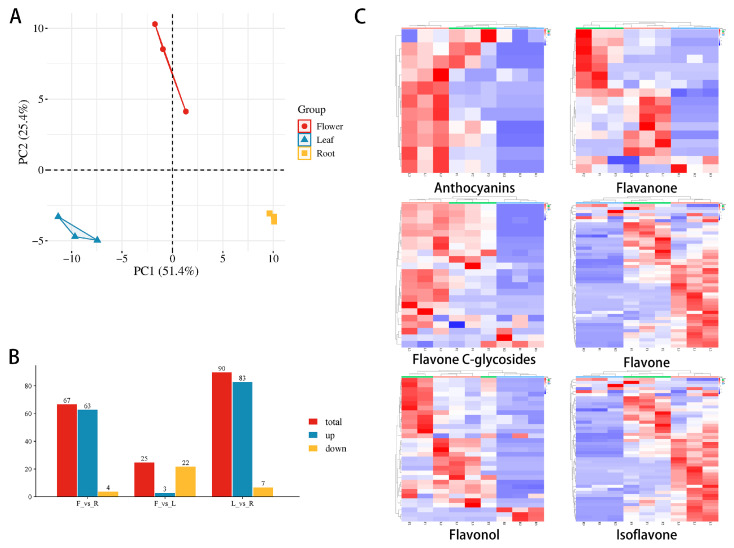
Analysis of Metabolite Accumulation Characteristics Across Three Tissues of *P. quinquefolius* (**A**) PCA of flavonoid metabolites in different tissues of *P. quinquefolius*. (**B**) Bar chart displaying the number of differentially accumulated metabolites among the tissues. (**C**) Clustering heatmap of flavonoid metabolites in different tissues of *P. quinquefolius*. Red indicates high abundance of metabolites, while blue represents relatively low abundance.

Liquid chromatography-mass spectrometry (UPLC-MS/MS) was used to analyze the composition of metabolites in the roots, leaves, and flowers of *P. quinquefolius*. PCA results showed distinct differences among the three tissues, with each biological replicate displaying strong correlation within groups (Figure 1A). Flowers and leaves exhibited more similar metabolic profiles, while roots displayed a distinct metabolic pattern. A total of 67 differentially accumulated metabolites (DAMs) were identified between flowers and roots, with 63 upregulated and 4 downregulated in flowers. Between flowers and leaves, 25 DAMs were identified, of which 3 were upregulated and 22 downregulated in flowers. When comparing leaves and roots, 90 DAMs were identified in roots, with 83 upregulated and 7 downregulated (Figure 1B).

A total of 141 flavonoid metabolites were identified across the roots, leaves, and flowers, including 47 flavones, 31 flavonols, 22 C-glycosylflavones, 17 flavanones, 13 isoflavones, and 11 anthocyanins (Table S2). Clustering analysis of the accumulation of six types of flavonoid metabolites across different tissues (Figure 1C, The respective clustering analysis plots are shown in Figures S1–S6.) revealed significant differences in flavonoid metabolite levels between leaves and roots. Specifically, flavonols and anthocyanins were highly enriched in flowers, while flavones and C-glycosylflavones accumulated predominantly in leaves. In contrast, the roots exhibited a relatively low accumulation of flavonoids, with the exception of specific compounds such as tricin 7-*O*-acetylglucoside. The accumulation of flavonoid metabolites in the underground part (roots) was markedly lower than that in the aboveground part, with only a few flavonoid compounds exhibiting higher accumulation in the roots, which may be associated with the growth and development characteristics of the roots.

### 2.2. Identification of Differentially Expressed Genes (DEGs) and Transcriptomic Analysis Among Three Tissues

**Figure 2:**
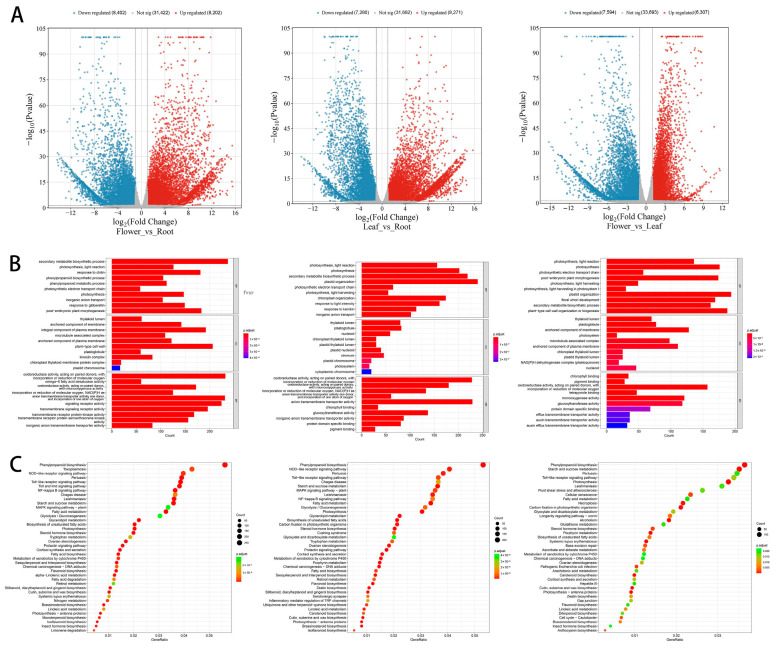
Analysis of DEGs Among Three Tissues of *P. quinquefolius* (**A**) Volcano plots of DEGs in comparisons between Flower_vs._Root, Flower_vs._Leaf, and Leaf_vs._Root. (**B**) GO annotation of DEGs. (**C**) KEGG enrichment analysis of DEGs.

To further investigate the molecular basis of flavonoid biosynthesis, transcriptome analysis was conducted to identify DEGs across roots, leaves, and flowers of *P. quinquefolius*. The number of DEGs among these three tissues is presented in Figure 2A. In the flower vs. root comparison, 8402 genes were downregulated and 8202 were upregulated in flowers relative to roots. In the leaf vs. root comparison, 8271 DEGs showed higher expression in leaves than in roots, while 7280 DEGs had lower expression. In the flower vs. leaf comparison, 6307 genes were upregulated and 7594 were downregulated in flowers.

To understand their biological functions and gene interactions, DEGs involved in metabolic pathways across different tissues were identified. Pairwise comparisons were conducted between the roots, flowers, and leaves of *Panax quinquefolius*, classifying DEGs into 30 functional groups. The three main categories of Gene Ontology (GO) terms are “Biological Process”, “Cellular Component”, and “Molecular Function” (Figure 2B). In the “Biological Process” category for the flower vs. leaf (F_vs._L) comparison, genes were highly enriched in “plastid organization” and “plant-type cell wall organization or biogenesis”. For the “Cellular Component” category, the most enriched term was “anchored component of membrane”. Within “Molecular Function”, “oxidoreductase activity, acting on paired donors, with incorporation or reduction of molecular oxygen” was the most abundant term. In the flower vs. root (F_vs._R) comparison, “secondary metabolite biosynthetic process” was the most enriched term in “Biological Process”. The “Cellular Component” category showed significant enrichment in “plant-type cell wall” and “integral component of plasma membrane”. The “Molecular Function” terms with the highest enrichment were “oxidoreductase activity, acting on paired donors, with incorporation or reduction of molecular oxygen” and “one donor and incorporation of one atom of oxygen anion transmembrane transporter activity”. For the leaf vs. root (L_vs._R) comparison, “plastid organization” and “secondary metabolic biosynthetic process” were the most enriched terms within “Biological Process”, while “oxidoreductase activity, acting on paired donors, with incorporation of reduction of molecular oxygen” and “anion transmembrane transporter activity” were prominent in “Molecular Function”.

To further analyze the functions of DEGs across different tissues of *P. quinquefolius*, Kyoto Encyclopedia of Genes and Genomes (KEGG) enrichment analysis was performed (Figure 2C). The results indicated that among all DEGs, the pathway with the highest enrichment was “phenylpropanoid biosynthesis”, a metabolic activity related to plant development, with the most prominent enrichment observed in the flower vs. root (F_vs._R) comparison. Additionally, GO enrichment analysis showed that DEGs were primarily enriched in the “secondary metabolic process”. These findings suggest that these DEGs are mainly involved in the biosynthesis and metabolism of secondary metabolites, potentially regulating the synthesis of flavonoid compounds in *P. quinquefolius*.

### 2.3. Validation of DEGs in Flavonoid Biosynthesis of Panax quinquefolius by qRT-PCR

**Figure 3:**
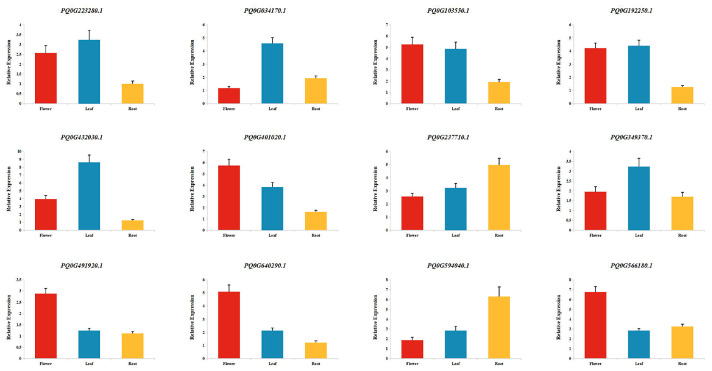
qRT-PCR Validation of DEGs randomly selected from three tissues of *P. quinquefolius*.

To validate the transcriptome data, qRT-PCR analysis was performed on DEGs related to flavonoid biosynthesis across three tissues of *P. quinquefolius*. As shown in Figure 3, the expression trends of all selected genes were consistent between qRT-PCR and RNA-seq data (Figure S7), thereby confirming the reliability of the transcriptomic data.

### 2.4. Correlation Analysis of Transcriptomic and Metabolomic Data

Correlation analysis was conducted to explore potential regulatory networks between genes and metabolites. Transcriptomic and metabolomic data revealed significant differences in metabolite accumulation and gene expression between the aerial tissues (leaves and flowers) and the underground tissue (roots) of *P. quinquefolius*. Therefore, transcriptomic data from the leaves, representing the aerial parts, and the roots, representing the underground parts, were selected for comparison, with 18,946 highly expressed genes (FPKM > 5) and 141 metabolites analyzed for correlations.

**Figure 4:**
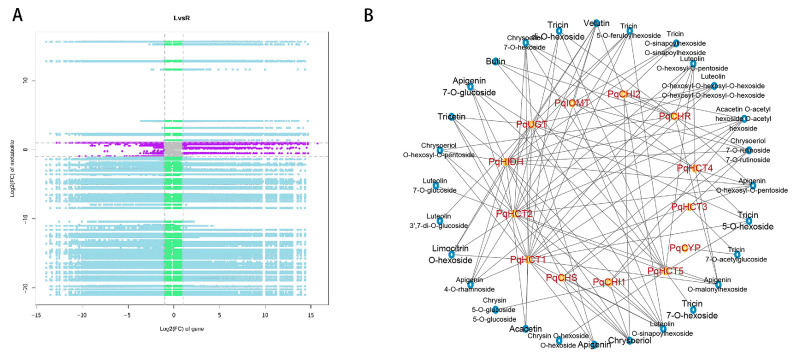
Integrated metabolomic and transcriptomic analysis across three tissues of *P. quinquefolius,* (**A**) Nine-quadrant plot of genes and metabolites. (**B**) Correlation analysis of DEGs and DAMs selected from the third and seventh quadrants.

As shown in the nine-quadrant plot (Figure 4A), genes and metabolites in the fifth quadrant, representing non-differentially expressed elements, were relatively few. Analysis of the third and seventh quadrants identified 13 DEGs significantly correlated with 27 differentially accumulated metabolites (Table S3). Furthermore, a single metabolite can be regulated by multiple genes, while conversely, a single gene can be regulated by multiple metabolites.

Based on the results from the third and seventh quadrants, a correlation network analysis was conducted between the identified DEGs and DAMs (Figure 4B), which revealed that the UDP-glycosyltransferases (UGTs) gene positively regulates the biosynthesis of luteolin 3′,7-di-*O*-glucoside. Among the three tissues of *P. quinquefolius*, chrysoeriol 7-*O*-acetyl-hexoside is exclusively present in roots, where it is associated with the regulatory activity of cytochrome CYP (Cytochrome P450) and the transferase HCT (Hydroxycinnamate: CoA ligase). The metabolites isoschaftoside, Limocitrin *O*-hexoside, Apigenin *O*-hexosyl-*O*-pentoside, Apigenin *O*-malonylhexoside, Tricin di-*O*-hexoside, Tricin 5-*O*-feruloylhexoside, Luteolin *O*-hexosyl-*O*-hexosyl-*O*-hexoside, Chrysoeriol *O*-hexosyl-*O*-pentoside, Chrysoeriol, Apigenin, Apigenin 4-*O*-rhamnoside, Luteolin 7-*O*-glucoside, Tricetin and Butin accumulated in leaves and flowers but were absent in roots. Other flavonoid metabolites were detected in roots, leaves, and flowers, although their accumulation in roots was consistently lower than in the aerial parts. This suggests that the high expression levels of these genes in leaves and flowers may contribute to the increased metabolite accumulation in these tissues, resulting in a greater diversity and concentration of flavonoids in the aerial parts compared to the roots. These findings provide new insights into the biosynthetic mechanisms of flavonoid compounds in different tissues of *P. quinquefolius*.

### 2.5. Changes in Genes and Metabolites in the Flavonoid Biosynthesis Regulatory Network

**Figure 5:**
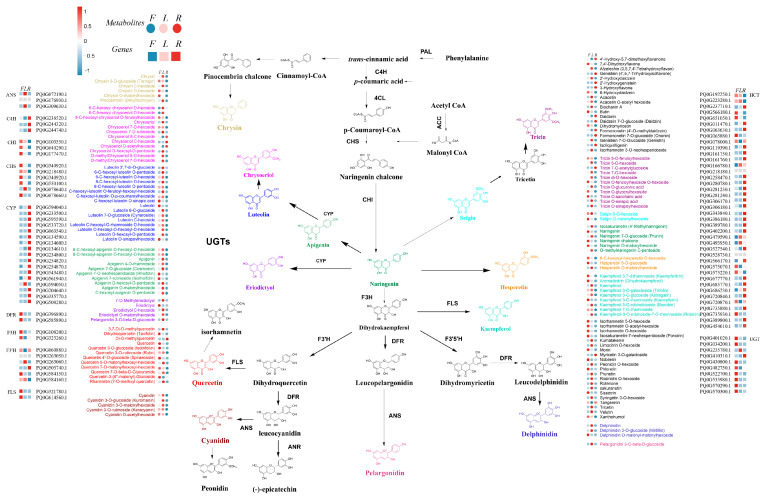
Flavonoid biosynthesis pathway in *P. quinquefolius.* Square heatmaps represent the expression levels of differentially expressed key enzyme genes across different tissues, while circular heatmaps represent the accumulation levels of differentially accumulated flavonoid metabolites in various tissues.

To analyze the differences in flavonoid compounds across different tissues of *P. quinquefolius*, metabolomic and transcriptomic data were integrated. A pathway map for flavonoid biosynthesis was constructed based on the detected metabolites (Figure 5), and DEGs involved in flavonoid biosynthesis, including Cinnamate-4-hydroxylase (C4H), Chalcone Synthase (CHS), Chalcone Isomerase (CHI), Flavanone 3-Hydroxylase (F3H), CYP, UGT, and Flavonol Synthase (FLS), were identified (Table S4). Apigenin, luteolin, and isovitexin accumulated at higher levels in flowers and leaves than in roots. Their corresponding glycone also showed lower accumulation in roots compared to leaves and flowers, correlating with the higher expression levels of UGTs in the aerial tissues. Among the 11 flavonoid compounds derived from tricin through UGT activity, only tricin 7-*O*-acetylglucoside accumulated at higher levels in roots than in leaves and flowers, and its corresponding *UGT* (Pq0G410310.1) showed the highest expression in roots. The remaining flavonoid compounds exhibited higher accumulation in leaves and flowers, which may explain the overall higher flavonoid content in these tissues. *CHIs* (Pq0G103550.1, Pq0G177470.1, Pq0G640290.1) and its downstream genes *F3H* (Pq0G325260.1) and *FLS* (Pq0G614560.1) had the highest expression in flowers. In contrast, *C4Hs* (Pq0G238520.1, Pq0G244320.1, Pq0G244740.1) and all annotated pathway genes of the *CYPs* family exhibited distinct expression patterns from other genes in this pathway, with the highest expression levels observed in the roots. The distribution patterns of differentially accumulated flavonoid compounds were consistent with the expression patterns of the related genes. For example, *FLS* (Pq0G614560.1) exhibited higher expression levels in the aerial parts than in the underground parts, and its downstream metabolites, such as quercetin and kaempferol, also accumulated at higher levels in the aerial parts than in the underground parts.

These results indicate the existence of a complex regulatory network linking the accumulation of secondary metabolites and gene expression levels. Notably, key flavonoid biosynthetic genes, including PAL, CHS, and FLS, exhibited significantly higher expression levels in flowers and leaves compared to roots, aligning with the metabolic differences observed among these three tissues. Furthermore, comparative analysis with other *Panax* species, such as *Panax ginseng* and *Panax notoginseng*, revealed that while *P. quinquefolius* shares conserved regulatory mechanisms in flavonoid biosynthesis, it also exhibits species-specific regulatory characteristics. This finding underscores the necessity for further investigation. Therefore, future studies should focus on the functional validation of these key genes to elucidate their precise roles in flavonoid biosynthesis in *P. quinquefolius*.

### 2.6. Functional Validation of Flavonoid-Related TFs and UGTs in P. quinquefolius

**Figure 6:**
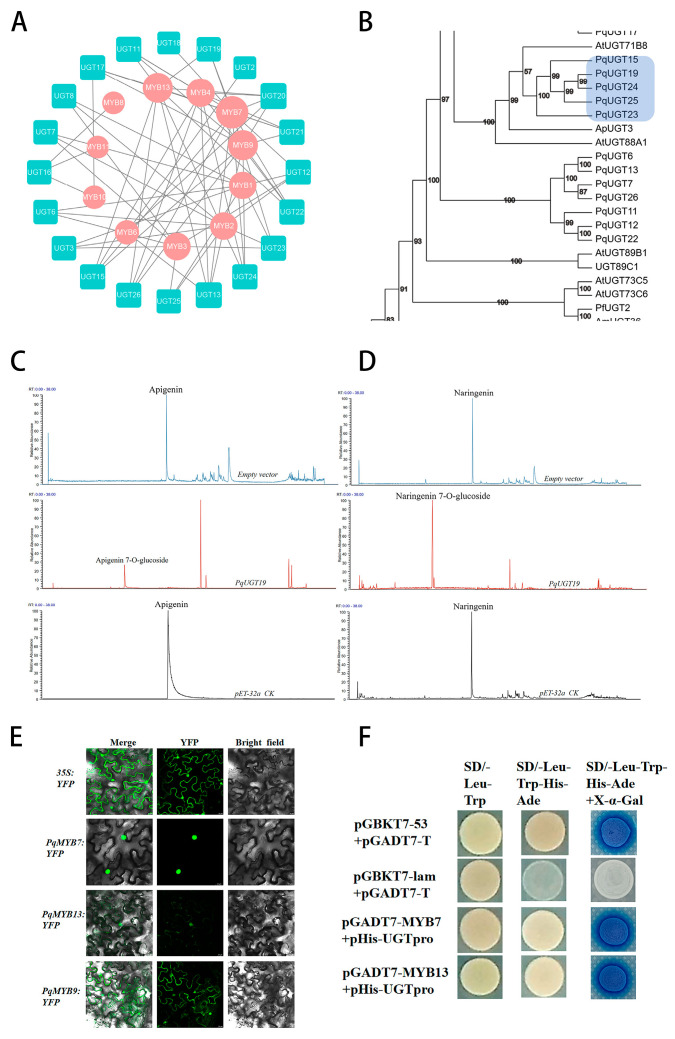
Functional validation of *PqUGTs* and *PqMYBs* in *P. quinquefolius*. (**A**) Correlation analysis of 27 highly expressed *PqUGTs* and 13 *PqMYBs*. (**B**) Phylogenetic analysis of the amino acid sequences of selected *PqUGTs* and *UGT* genes from various plant species, with the selected candidate genes highlighted in the blue box. (**C**) UPLC-MS chromatogram showing the catalytic product of *PqUGT19* with apigenin as the substrate. (**D**) UPLC-MS chromatogram showing the catalytic product of *PqUGT19* with naringenin as the substrate. (**E**) Subcellular localization of *PqMYB7*, *PqMYB9*, and *PqMYB13* in vivo. (**F**) Yeast one-hybrid assay demonstrating the interaction of *PqMYB7* and *PqMYB13* with the *PqUGT19* promoter.

A correlation analysis was conducted using 26 highly expressed *PqUGT*s and 13 *PqMYB*s identified in the flavonoid biosynthetic pathway (Table S5, Figure 6A). To further select candidate *UGT*s, a phylogenetic tree was constructed based on previously reported flavonoid *UGTs* and *PqUGTs* with high degree values from the correlation analysis (Figure S9). This analysis revealed clustering of certain glycosyltransferases from *P. quinquefolius* with those of other species (Figure 6B).

To confirm the functional role of these glycosyltransferases in flavonoid glycosylation, five candidate genes were selected for in vitro validation (Table S6). The gene sequences of the five *PqUGTs* are shown in Table S7. Prokaryotic expression vectors were constructed, and recombinant proteins were expressed and purified. The concentrated crude proteins were subsequently tested for in vitro enzymatic activity using flavonoid substrates. The results (Figure 6C,D) indicated that only *PqUGT19* could catalyze apigenin and naringenin to form apigenin-7-*O*-glucoside and naringenin-7-*O*-glucoside, confirming that *PqUGT19* functions as a flavonoid glycosyltransferase.

To investigate the subcellular localization of *PqMYB7*, *PqMYB9*, and *PqMYB13*, their encoding genes were fused with the PHB-YFP vector and subsequently transformed into tobacco leaves. We utilized the Leica STELLARIS 5 laser confocal microscope. In the software settings, we assigned the YFP marker to the green channel to display green fluorescence during observation. The merged images display overlay fields: YFP fluorescence images under 514 nm excitation indicate green fluorescent protein expression, while bright-field images reveal cellular structures. The results indicated that, in contrast to the control vector (YFP), which was distributed throughout both the nucleus and cytoplasm, *PqMYB7*-YFP and *PqMYB13*-YFP exhibited fluorescence exclusively in the nucleus. In addition, *PqMYB9*-YFP displayed fluorescence in both the nucleus and cytoplasm. These findings indicate that *PqMYB7* and *PqMYB13* are localized in the nucleus, supporting their predicted role as TFs (Figure 6E).

The promoter of *PqUGT19* was analyzed using the PlantCARE database, which identified cis-elements for MYB TFs, suggesting that *PqUGT19* may be regulated by MYB TFs. Promoter-binding elements for MYBs were also identified in the promoter region of *UGT19* (Figure S8). The yeast one-hybrid assay results further indicated that *PqMYB7* and *PqMYB13* can bind to the promoter elements of *PqUGT19* (Figure 6F). This observation is consistent with related studies on *Citrus aurantium L.* (sour orange), where MYB transcription factors regulate the expression of UGT genes, participate in the biosynthesis pathway of flavonoid compounds, and play a key role in the developmental process of sour orange. Taken together, these findings suggest that *PqMYB7* and *PqMYB13* regulate the expression of *PqUGT19* in *P. quinquefolius* by binding to its promoter, thereby influencing the biosynthesis of apigenin-7-*O*-glucoside and naringenin-7-*O*-glucoside.

## 3. Discussion

*P. quinquefolius* harbors a diverse repertoire of metabolites and an intricate gene expression regulatory network, with a distinct regulatory emphasis on flavonoid biosynthesis. To elucidate the factors contributing to variations in gene expression and metabolite accumulation within the flavonoid biosynthetic pathway, we analyzed gene-metabolite correlations in distinct tissues of *P. quinquefolius*. Given that gene expression is influenced by various internal and external factors [34], transcriptome analysis alone is insufficient to fully elucidate the patterns of metabolite accumulation [35,36,37]. As the end products of biosynthetic processes, metabolites are regulated by numerous endogenous and exogenous factors [38]. Therefore, integrating transcriptomic and metabolomic approaches can aid in identifying key functional genes involved in critical metabolic pathways or molecular mechanisms [39,40]. For example, combined transcriptomic and metabolomic analyses have successfully identified key genes in flavonoid biosynthesis in blackberry [41] and candidate genes involved in phenolic acid biosynthesis in *Cyclocarya paliurus* leaves [42].

### 3.1. Transcriptome Analysis and qRT-PCR Validation

In this study, transcriptome analysis was initially conducted to identify DEGs in different tissues of *P. quinquefolius*. The reliability of the transcriptomic data was verified through qRT-PCR. A total of 15,551 DEGs were identified in the root vs. leaf comparison, while 16,604 DEGs were detected in the root vs. flower comparison. *P. quinquefolius* exhibited a significantly higher number of DEGs compared to other plant species, such as Tartary buckwheat [43] and *Camellia lanceoleosa* [44]. This difference may be attributed to its polyploid genome, complex secondary metabolic pathways, and distinct physiological adaptations between underground (roots) and aerial (leaves and flowers) tissues [45,46,47].

### 3.2. Metabolite Profiling

Furthermore, metabolomic analysis quantified metabolites in roots, flowers, and leaves of *P. quinquefolius* [48,49]. Metabolite profiling demonstrated tissue-specific accumulation patterns, with most flavonoid compounds exhibiting higher concentrations in leaves and flowers than in roots. A limited number of flavonoids, however, accumulated at higher levels in roots.

### 3.3. Flavonoid Biosynthesis Pathway and Tissue-Specific Accumulation

By integrating metabolomic and transcriptomic data, this study examined the differences in flavonoid content and gene expression patterns across various tissues of *P. quinquefolius*. Based on the detected differentially accumulated metabolites and DEGs, a flavonoid biosynthesis pathway map was constructed, identifying 13 types key DEGs in this pathway, including *OMTs*, *CHIs*, *HCTs*, *HIDHs*, *UGTs*, *CHRs*, *CHSs*, and *CYPs*. Among these pathway genes, *UGT* (Pq0G410310.1) expression was significantly higher in leaves and flowers, which may explain the elevated accumulation of flavonoid glycosides in these tissues. The distribution pattern of flavonoid compounds across different tissues was consistent with the expression patterns of the related genes. This result aligns with previous findings [50], which demonstrated that flavonoid compounds are widely distributed in various plant tissues, with significantly higher accumulation in the aerial parts (particularly leaves and flowers) compared to roots in most species. Based on these findings, it is hypothesized that the differential expression of flavonoid-modifying genes may play a crucial role in the tissue-specific accumulation patterns of these metabolites. Flavonoid compounds have been shown to effectively prevent and reduce potential root damage in plants caused by various diseases and pests [51,52]. Studies indicate that isoflavonoids in pea roots, such as biochanin A, maackiain, and genistein, exhibit antifungal activity against soil fungi in the pea rhizosphere and possess antioxidant properties [53]. This may explain the high concentrations of certain flavonoid compounds in roots.

### 3.4. Regulation of Flavonoid Biosynthesis by TFs

Additionally, these tissue-specific metabolite variations may be driven by the regulation of TFs and structural genes. The biosynthesis of flavonoids involves a complex transcriptional regulatory network composed of multiple TFs, which can bind to genes encoding key enzymes in the flavonoid biosynthetic pathway, thereby influencing the entire pathway. The MYB TFs family plays a crucial regulatory role in the biosynthesis of flavonoid compounds, primarily controlling the biosynthesis of flavonoids, cellulose, and lignin, as well as the formation of secondary cell walls in plants [54]. MYB TFs can directly influence the expression of key flavonoid genes or modulate the process by interacting with other TFs. For example, in *A. thaliana*, *MYB111* positively regulates the expression of *CHS*, *F3H*, and *FLS1* genes, enhancing the plant’s salt stress tolerance [55]. In *Panax ginseng*, the R2R3-MYB transcription factor *PgTT2* regulates proanthocyanidin biosynthesis through interaction with the *PgTTG1* protein [56]. In *Scutellaria baicalensis*, *SbMYB3* regulates flavone synthase II-2 (SbFNSII-2), thereby increasing the levels of baicalin, baicalein, and wogonin [57]. MYB TFs modulate the accumulation of flavonoids in *Panax notoginseng* by regulating the expression of genes involved in flavonoid biosynthesis [58].

### 3.5. Glycosylation of Flavonoids by UGTs

The glycosylation of flavonoids is primarily catalyzed by UGTs, with flavonoids in plants typically existing in glycoside forms. Previous studies have demonstrated that celery *UGT94AX1* can catalyze the biosynthesis of the flavone glycoside apiin [59]. In this study, the PqUGTs identified through screening were found, by in vitro assays, to catalyze the glycosylation of quercetin and luteolin at the 7-OH position. BLAST, version 2.12.0. https://blast.ncbi.nlm.nih.gov/Blast.cgi accessed on 25 February 2025) analysis revealed that *PqUGT19* exhibits high sequence similarity with members of the UGT71A family. This family is known to participate in the glycosylation of flavonoid compounds. For example, a novel 7-*O*-glycosyltransferase *CmGT* from *Cucurbita moschata* was cloned, which could efficiently convert luteolin into luteolin-7-*O*-glucoside under optimal conditions [60]. This enzyme family is involved in the glycosylation of flavonoid compounds, playing a critical role in plant defense, hormone regulation, and the biosynthesis of secondary metabolites, which enables plants to adapt to fluctuating environmental conditions [61]. It has been extensively characterized in plants such as *A. thaliana* [62] and *Panax notoginseng* [63]. Consequently, *PqUGT19* was classified as *PqUGT71A1*.

### 3.6. Regulatory Network of UGTs and MYB Transcription Factors in Flavonoid Accumulation

**Figure 7:**
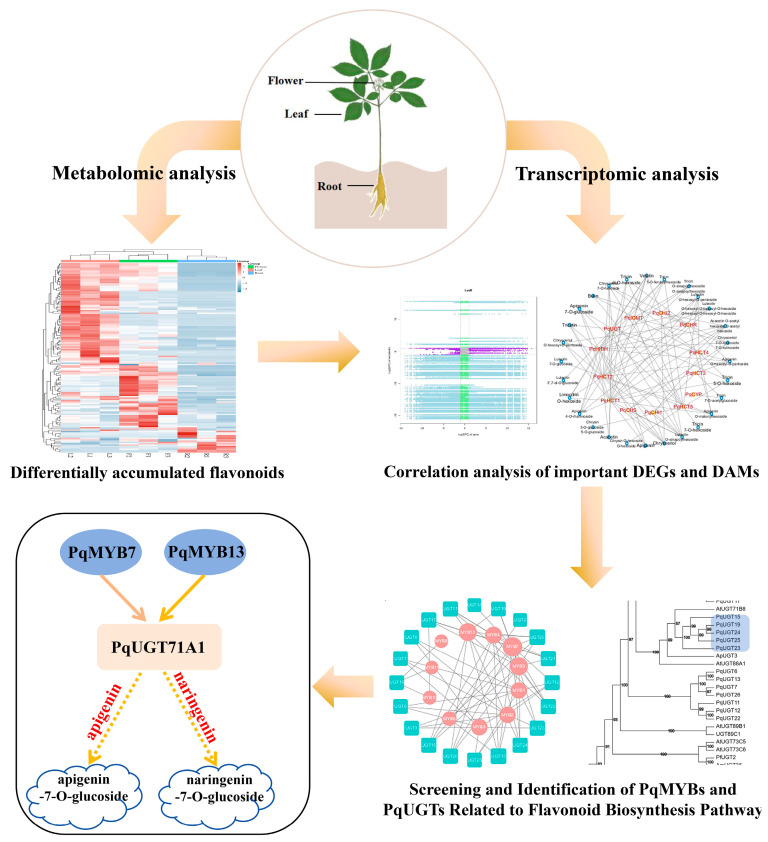
Schematic representation of the MYB-UGT regulatory axis in flavonoid biosynthesis in *P. quinquefolius*.

UGTs and MYB transcription factors not only function independently but may also work together through a complex regulatory network to regulate the accumulation of flavonoid compounds in different plant tissues. The role of the MYB-UGT regulatory axis in the biosynthesis of flavonoid compounds in *P. quinquefolius* is shown in Figure 7. Additionally, in blueberries (*Vaccinium* spp.), *VcMYB* forms a regulatory network with abscisic acid response element binding factors, bHLH, and flavonoid biosynthetic genes to control the accumulation of flavonoid metabolites in drought-induced blueberry leaves [64]. Therefore, the flavonoid accumulation patterns in the roots and leaves of *P. quinquefolius* may be coordinately regulated by the expression patterns of MYB transcription factors and UGT genes. MYB TFs regulate the expression of UGT genes, thereby affecting the distribution of flavonoid compounds in different plant tissues. Furthermore, in our previous study [65], some R2R3-MYB TFs were hypothesized to be involved in flavonoid biosynthesis in *P. quinquefolius*. In this study, *PqMYB7* and *PqMYB13* from the C17 subfamily of the previously identified MYB family were further studied. Validation experiments revealed that these TFs can regulate the expression of *PqUGT71A1*, which is associated with corresponding changes in flavonoid content across the roots, leaves, and flowers of *P. quinquefolius*. The functional characterization of UGTs and TFs in this study provides a foundation for further research into the regulatory mechanisms of flavonoid biosynthesis in *P. quinquefolius*.

## 4. Materials and Methods

### 4.1. Plant Materials and Sample Collection

To perform transcriptomic and metabolomic analyses of *P. quinquefolius* roots, leaves, and flowers. The *P. quinquefolius* plant material used in this study was collected from Wanliang Township, Fusong County (42°26′9.22″ N, 127°17′21.00″ E) on 9 June 2018, a period characterized by stable plant growth. The specimens were identified as 4-year-old *P. quinquefolius* by Professor Tian Yixin from Jilin Agricultural University. Three plants of similar size and growth status were selected for sampling. From each plant, the main roots (2 cm below the rhizome), leaves, and flower buds were thoroughly washed, cut into small pieces, flash-frozen in liquid nitrogen, and divided into two aliquots for storage at −80 °C until further analysis.

### 4.2. Sample Preparation and Extraction

To extract flavonoid compounds for UPLC-MS/MS analysis. Samples (100 mg each) were freeze-dried using a vacuum freeze dryer. The dried samples were then ground using a bead mill (MM400, RETSCH) at 30 Hz for 1.5 min. The powdered samples were suspended in 70% methanol (1000 μL) and stored at 4 °C overnight. During extraction, the samples were vortexed three times and centrifuged at 4 °C at 10,000× *g* for 15 min. The supernatant was then filtered through a 0.22 μm pore size filter membrane (SCAA-104, ANPEL, Shanghai, China) and used for UPLC-MS/MS analysis.

### 4.3. UPLC-MS/MS Determination of Flavonoid Compounds

To detect flavonoid compounds in *P. quinquefolius*, the filtered supernatant from the sample extraction was analyzed using a UPLC-MS/MS system (HPLC: Shim-pack UFLC SHIMADZU CBM30A; MS: Applied Biosystems 6500 QTRAP). The liquid chromatography conditions were as follows: (i) Column: Waters ACQUITY UPLC HSS T3 C_18_, 1.8 μm, 2.1 mm × 100 mm; (ii) Mobile phases: ultrapure water with 0.04% acetic acid as the aqueous phase, and acetonitrile with 0.04% acetic acid as the organic phase; (iii) Elution gradient: 0 min, water/acetonitrile (95:5, *v*/*v*); 11.0 min, 5:95 (*v*/*v*); 12.0 min, 5:95 (*v*/*v*); 12.1 min, 95:5 (*v*/*v*); 15.0 min, 95:5 (*v*/*v*); (iv) Flow rate: 0.4 mL/min; column temperature: 40 °C; injection volume: 5 μL.

### 4.4. Identification and Quantification of Flavonoid Metabolites

To analyze the accumulation differences of flavonoid metabolites between different tissues of *P. quinquefolius*, the acquired mass spectrometry data were processed using Analyst software for quality control of pooled samples. Qualitative analysis of the widely targeted metabolomics results was performed based on public databases, a custom-built database (MWDB, Metware Database), and commercially purchased standards. Peak detection and annotation were conducted using in-house R scripts and databases. Variable importance in projection (VIP) values of the first principal component in OPLS-DA analysis were obtained to visualize group separation and identify metabolites with significant changes. The VIP values summarize each variable’s contribution to the model, with VIP > 1, fold change ≥ 2 or ≤0.5, and *p* < 0.05 indicating DAMs.

### 4.5. RNA Extraction and Transcriptome Sequencing

To analyze the transcriptome data differences between different tissues of *P. quinquefolius*. Total RNA was extracted from the nine above mentioned samples using the RNeasy Plant Mini Kit (Qiagen, Germany), following the manufacturer’s instructions. RNA quality was assessed prior to library construction, which was performed using synthesis-based techniques to create complementary DNA (cDNA) libraries. Sequencing was conducted on the PacBio Sequel and Illumina HiSeq 2000 high-throughput platforms. The clean reads we reported previously [66] were aligned to the *P. quinquefolius* genome [67] using the workflow as reported [68]. Gene expression quantification was based on FPKM values (Fragments Per Kilobase of transcript per Million mapped reads). Differential expression analysis was conducted using the DESeq R, version 1.30.1 (https://bioconductor.org/packages/release/bioc/html/DESeq2.html accessed on 25 February 2025) package. Genes with adjusted *p* < 0.05 were considered significantly differentially expressed. Hierarchical clustering analysis was performed on DEGs to assess gene expression patterns. GO enrichment and KEGG pathway enrichment analyses of differentially expressed genes were conducted using R with a hypergeometric test.

### 4.6. Quantitative Real-Time Polymerase Chain Reaction (qRT-PCR) Analysis

To validate the reliability of the transcriptomic data, twelve flavonoid-related DEGs were selected for qRT-PCR. The primers used were synthesized by Sangon Biotech (Shanghai, China) and their sequences are listed in Table S1. Total RNA was extracted from the samples using Esippur Plana Kit (Transon Bioteh, Beijing, China) and Renas-Friednasyi (Transon Bioteh) to eliminate DNA contamination. The concentration and quality of the RNA samples were assessed using a NanoPhotometer N50 (Implen, GER, München, Germany). Subsequently, the Perfect-Start Uni qRT-PCR Kit (TransGen Biotech) was used to reverse transcribe RNA into cDNA. Two-step qRT-PCR kit was used for analysis, and the reaction procedure is as follows: predenaturation 94 °C, 30 s; denature 94 °C, 5 s; annealing temperature 60 °C, 30 s; 45 cycles. The reaction system is 20 µL: 2 × PerfectStart^®^ Green qPCR SuperMix 10 µL, F, R primers all 1 µL, cDNA 1 µL, filled to 20 µL with ddH_2_O. The *P. quinquefolius GAPDH* gene was used as the internal reference. The expression level of the corresponding genes was calculated using the 2^−ΔΔCT^ method.

### 4.7. Correlation Analysis of Transcriptome and Metabolome

Through the integrated analysis of transcriptomic and metabolomic data, key *PqMYBs* and *PqUGTs* involved in the regulation of flavonoid biosynthesis were identified. Functional validation experiments, such as subcellular localization and yeast one-hybrid assays, were subsequently performed to further investigate the interaction between *PqMYBs* and *PqUGTs* and their roles in the regulation of flavonoid biosynthesis in *P. quinquefolius*.

The Pearson correlation coefficient test was used to calculate the correlation between DEGs and DAMs related to the flavonoid biosynthesis pathway in *P. quinquefolius*. A Pearson correlation coefficient (PCC) ≥ 0.9 and *p*-value ≤ 0.05 were selected as thresholds, and results were visualized using nine-quadrant plots. Key genes and metabolites were identified based on PCC > 0.9 and |log_2_(FC)| > 2 through correlation network analysis.

The subcellular localization method was as follows: The selected *PqMYBs* were inserted into the PHB-YFP vector. The correctly sequenced *PqMYB*-YFP recombinant plasmid was then transferred into the Agrobacterium tumefaciens strain GV 3101. The GV 3101 strain containing the recombinant plasmid was used to infect tobacco leaves, with the empty PHB-YFP vector serving as the control. The samples were observed using the Leica STELLARIS 5 laser confocal microscope.

The yeast one-hybrid assay was conducted to verify the DNA binding of *PqMYBs* to the promoter regions of *PqUGTs* by constructing the *PqMYB*-pGADT7 and MYB-binding site-p53-His2 vectors. First, the pGADT7 vector was digested with EcoR I and BamH I, and the *PqMYBs* fragment was recombined with the vector and transformed into *Escherichia coli*. Correct recombinant plasmids were selected and verified. Next, a DNA fragment containing MYB-binding sites was synthesized and ligated into the p53-His2 vector, followed by sequencing verification. Finally, the constructed plasmids were co-transformed into yeast competent cells. After culturing and colorimetric reaction, the interaction between *PqMYBs* and the promoter regions of *PqUGTs* was validated.

## 5. Conclusions

By integrating transcriptomic and metabolomic analyses, this study systematically elucidates the tissue-specific accumulation patterns of flavonoids in *P. quinquefolius* and their underlying molecular regulatory networks. A total of 141 flavonoid metabolites were identified in *P. quinquefolius* for the first time, revealing significant enrichment of flavones, flavonols, and C-glycosylflavones in aerial tissues (leaves and flowers). Additionally, a distinct tissue-specific accumulation pattern was observed for certain glycosylated compounds, such as tricin 7-*O*-acetylglucoside, which was predominantly accumulated in roots. Transcriptomic analysis identified 15,551–18,946 DEGs, highlighting the phenylpropanoid biosynthetic pathway as a key regulatory hub and uncovering a coordinated interaction between UGTs and MYB TFs in flavonoid glycosylation through co-expression network analysis.

Functional validation experiments demonstrated, for the first time, that *PqUGT71A1* catalyzes the glycosylation of apigenin and naringenin at the 7-OH position. Furthermore, *PqMYB7* and *PqMYB13* were found to directly bind to the promoter region, activating *PqUGT71A1* expression and thereby establishing the MYB-UGT regulatory axis. These findings not only address a critical gap in understanding the spatial dynamics of flavonoid metabolism in *P. quinquefolius* but also provide a novel framework for elucidating the tissue-specific regulation of secondary metabolite biosynthesis in medicinal plants.

This study lays a theoretical foundation for metabolic engineering and the optimization of bioactive compounds in medicinal plants. Targeted regulation of the MYB-UGT axis enables precise modulation of flavonoid biosynthesis in specific tissues of *P. quinquefolius*. For instance, gene editing or overexpression strategies could be employed to enhance the production of high-value flavonoids in aerial tissues or to regulate the accumulation of root-specific glycosides for the development of novel medicinal compounds.

Moreover, the integrative analytical approach established in this study is broadly applicable to other medicinal plants, facilitating the elucidation and utilization of natural product biosynthetic pathways. Future research could further explore the ecological functions of flavonoids in *P. quinquefolius*, such as their roles in stress resistance and symbiotic signaling, through multi-omics approaches to gain deeper insights into the dynamic interplay between metabolites and gene regulation.

## Data Availability

Data are contained within the article.

## Supplementary Materials

The following supporting information can be downloaded at: https://www.mdpi.com/article/10.3390/ijms26062669/s1.

## References

[1] Kim D.H. (2012). Chemical Diversity of Panax ginseng, Panax quinquifolium, and Panax notoginseng. J. Ginseng Res..

[2] Qu C., Li B., Lai Y., Li H., Windust A., Hofseth L.J., Nagarkatti M., Nagarkatti P., Wang X.L., Tang D. (2015). Identifying panaxynol, a natural activator of nuclear factor erythroid-2 related factor 2 (Nrf2) from American ginseng as a suppressor of inflamed macrophage-induced cardiomyocyte hypertrophy. J. Ethnopharmacol..

[3] Shao Z.-H., Xie J.T., Vanden Hoek T.L., Mehendale S., Aung H., Li C.-Q., Qin Y., Schumacker P.T., Becker L.B., Yuan C.S. (2004). Antioxidant effects of American ginseng berry extract in cardiomyocytes exposed to acute oxidant stress. Biochim. Biophys. Acta.

[4] Yun T.K., Yun Y.S., Han I.W. (1983). Anticarcinogenic effect of long-term oral administration of red ginseng on newborn mice exposed to various chemical carcinogens. Cancer Detect. Prev..

[5] Dodd G.F., Williams C.M., Butler L.T., Spencer J.P.E. (2019). Acute effects of flavonoid-rich blueberry on cognitive and vascular function in healthy older adults. Nutr. Healthy Aging.

[6] Chang F., Jia F., Lv R., Guan M., Jia Q., Sun Y., Li Z. (2022). Effects of American Ginseng Cultivation on Bacterial Community Structure and Responses of Soil Nutrients in Different Ecological Niches. J. Microbiol. Biotechnol..

[7] Qiang B., Miao J., Phillips N., Wei K., Gao Y. (2020). Recent Advances in the Tissue Culture of American Ginseng (*Panax quinquefolius*). Chem. Biodivers..

[8] Roleira F.M.F., Varela C.L., Costa S.C., Tavares-da-Silva E.J. (2018). Phenolic Derivatives From Medicinal Herbs and Plant Extracts: Anticancer Effects and Synthetic Approaches to Modulate Biological Activity. Stud. Nat. Prod. Chem..

[9] Ververidis F., Trantas E., Douglas C., Vollmer G., Kretzschmar G., Panopoulos N. (2007). Biotechnology of flavonoids and other phenylpropanoid-derived natural products. Part I: Chemical diversity, impacts on plant biology and human health. Biotechnol. J..

[10] Wen W., Alseekh S., Fernie A.R. (2020). Conservation and diversification of flavonoid metabolism in the plant kingdom. Curr. Opin. Plant Biol..

[11] Yu W., Gong F., Xu H. (2024). Molecular Mechanism of Exogenous ABA to Enhance UV-B Resistance in *Rhododendron chrysanthum* Pall. by Modulating Flavonoid Accumulation. Int. J. Mol. Sci..

[12] Kuhn B.M., Nodzyński T., Errafi S., Bucher R., Gupta S., Aryal B., Dobrev P., Bigler L., Geisler M., Zažímalová E. (2017). Flavonol-induced changes in PIN2 polarity and auxin transport in the *Arabidopsis thaliana* rol1-2 mutant require phosphatase activity. Sci. Rep..

[13] Mughal N., Zhang X., Shoaib N., Deng J., Guo J., Zhang J., Yang W., Liu J. (2025). Screening of soybean antifungal isoflavones based on targeted metabolomics analysis. Food Chem..

[14] Yu Q., Liu C., Sun J., Ding M., Ding Y., Xu Y., He J., Li Q., Jin X. (2024). McWRKY43 Confers Cold Stress Tolerance in *Michelia crassipes* via Regulation of Flavonoid Biosynthesis. Int. J. Mol. Sci..

[15] Yun C.S., Yamamoto T., Nozawa A., Tozawa Y. (2008). Expression of Parsley Flavone Synthase I Establishes the Flavone Biosynthetic Pathway in *Arabidopsis thaliana*. Biosci. Biotechnol. Biochem..

[16] Li S., Deng B., Tian S., Guo M., Liu H., Zhao X. (2021). Metabolic and transcriptomic analyses reveal different metabolite biosynthesis profiles between leaf buds and mature leaves in *Ziziphus jujuba* mill. Food Chem..

[17] Yang X., Xu Q., Le L., Zhou T., Yu W., Wang G., Fu F.F., Cao F. (2023). Comparative histology, transcriptome, and metabolite profiling unravel the browning mechanisms of calli derived from ginkgo (*Ginkgo biloba* L.). J. For. Res..

[18] Meng X., Li G., Gu L., Sun Y., Li Z., Liu J., Wu X., Dong T., Zhu M. (2020). Comparative Metabolomic and Transcriptome Analysis Reveal Distinct Flavonoid Biosynthesis Regulation Between Petals of White and Purple *Phalaenopsis amabilis*. J. Plant Growth Regul..

[19] Parkhomchuk D., Borodina T., Amstislavskiy V., Banaru M., Hallen L., Krobitsch S., Lehrach H., Soldatov A. (2009). Transcriptome analysis by strand-specific sequencing of complementary DNA. Nucleic Acids Res..

[20] Zhang M., Wang W., Liu Q., Zang E., Wu L., Hu G., Li M. (2023). Transcriptome analysis of *Saposhnikovia divaricata* and mining of bolting and flowering genes. Chin. Herb. Med..

[21] Covington M.F., Maloof J.N., Straume M., Kay S.A., Harmer S.L. (2008). Global transcriptome analysis reveals circadian regulation of key pathways in plant growth and development. Genome Biol..

[22] Fiehn O., Kopka J., Dormann P., Altmann T., Trethewey R.N., Willmitzer L. (2000). Metabolite profiling for plant functional genomics. Nat. Biotechnol..

[23] Huang Y., Bian C., Huang Y., Hou W., Xue H., Xing Y., Zheng H., Yang Y., Kang T., Xu L. (2024). Targeted metabolomics analysis of three medicinal plants of the genus *Pulsatilla*. Med. Plant Biol..

[24] Sumner L.W., Mendes P., Dixon R.A. (2003). Plant metabolomics: Large-scale phytochemistry in the functional genomics era. Phytochemistry.

[25] Van Assche R., Broeckx V., Boonen K., Maes E., De Haes W., Schoofs L., Temmerman L. (2015). Integrating -Omics: Systems Biology as Explored Through *C-elegans* Research. J. Mol. Biol..

[26] Li Y., Fang J., Qi X., Lin M., Zhong Y., Sun L., Cui W. (2018). Combined Analysis of the Fruit Metabolome and Transcriptome Reveals Candidate Genes Involved in Flavonoid Biosynthesis in *Actinidia arguta*. Int. J. Mol. Sci..

[27] Wei Y., Zhang J., Qi K., Li Y., Chen Y. (2023). Combined analysis of transcriptomics and metabolomics revealed complex metabolic genes for diterpenoids biosynthesis in different organs of *Anoectochilus roxburghii*. Chin. Herb. Med..

[28] Zhang A., Zheng J., Chen X., Shi X., Wang H., Fu Q. (2021). Comprehensive Analysis of Transcriptome and Metabolome Reveals the Flavonoid Metabolic Pathway Is Associated with Fruit Peel Coloration of Melon. Molecules.

[29] Lai J., Li C., Zhang Y., Wu Z., Li W., Zhang Z., Ye W., Guo H., Wang C., Long T. (2023). Integrated Transcriptomic and Metabolomic Analyses Reveal the Molecular and Metabolic Basis of Flavonoids in *Areca catechu* L.. J. Agric. Food Chem..

[30] Yu J., Chen W., Wang D., Xu L., Wang X. (2023). Cytotoxic constituents from the leaves and stems of *Panax quinquefolius*. Nat. Prod. Res..

[31] Lim E.K. (2003). Evolution of substrate recognition across a multigene family of glycosyltransferases in *Arabidopsis*. Glycobiology.

[32] Wilson A.E., Wu S., Tian L. (2019). *Pg*UGT95B2 preferentially metabolizes flavones/flavonols and has evolved independently from flavone/flavonol UGTs identified in *Arabidopsis thaliana*. Phytochemistry.

[33] Liu X., Xu W., Li J., Feng X., Li C. (2024). Identification of a permissive glycosyltransferase UGT73AC11 for flavonoids glycosylation. Biochem. Eng. J..

[34] Chen Y., Zahavi E., Barak P., Umiel N. (1980). Effects of Salinity Stresses on Tobacco: I. The Growth of *Nicotiana tabacum* Callus Cultures under Seawater, NaCl, and Mannitol Stresses. Z. Für Pflanzenphysiol..

[35] Urrutia M., Blein-Nicolas M., Prigent S., Bernillon S., Deborde C., Balliau T., Maucourt M., Jacob D., Ballias P., Benard C. (2021). Maize metabolome and proteome responses to controlled cold stress partly mimic early-sowing effects in the field and differ from those of *Arabidopsis*. Plant Cell Environ..

[36] Niu Y., Muhammad N., Duan Y., Wang Q., Duan Y., Li J., Liu M., Liu P., Wang L. (2024). Transcriptome and metabolome reveal the molecular mechanism of quality formation in different planting areas of *Ziziphus jujuba Mill*. ‘Jingudazao’ fruit. Ind. Crops Prod..

[37] Huang X., Rong W., Zhang X., Gao Y., Zhou Y., Su J., Luo H., Chu G., Wang M. (2024). Transcriptome and metabolome analysis reveal the dynamic changes and biosynthesis pathways of alkaloids in *Sophora alopecuroides L*. under drought stress. Ind. Crops Prod..

[38] Peng J., Dong B.Z., Wang D., Zhao Y.J., Meng H.W., Zhou H.Y. (2022). Analysis of differential metabolites of sunflower induced by virulent *Verticillium dahlia V33* and hypovirulent *Gibellulopsis nigrescens Vn-1*. J. Phytopathol..

[39] Wang P., Zhong L., Yang H., Hou X., Wu C., Zhang R., Yu J., Cheng Y. (2022). Systematic transcriptomic and metabolomic analysis of walnut (*Juglans regia L.*) fruit to trace variations in antioxidant activity during ripening. Sci. Hortic..

[40] Chen X., Shi X., Ai Q., Han J., Wang H., Fu Q. (2022). Transcriptomic and metabolomic analyses reveal that exogenous strigolactones alleviate the response of melon root to cadmium stress. Hortic. Plant J..

[41] Wu Y., Zhang C., Huang Z., Lyu L., Li W., Wu W. (2022). Integrative analysis of the metabolome and transcriptome provides insights into the mechanisms of flavonoid biosynthesis in blackberry. Food Res. Int..

[42] Lin W., Li Y., Lu Q., Lu H., Li J. (2020). Combined Analysis of the Metabolome and Transcriptome Identified Candidate Genes Involved in Phenolic Acid Biosynthesis in the Leaves of *Cyclocarya paliurus*. Int. J. Mol. Sci..

[43] Hou S., Du W., Hao Y., Han Y., Li H., Liu L., Zhang K., Zhou M., Sun Z. (2021). Elucidation of the Regulatory Network of Flavonoid Biosynthesis by Profiling the Metabolome and Transcriptome in Tartary Buckwheat. J. Agric. Food Chem..

[44] Song Q., Gong W., Yu X., Ji K., Chang Y., Wang L., Yuan D. (2022). Integrative analysis of the metabolome and transcriptome provides novel insights into the mechanisms of flavonoid biosynthesis in *Camellia lanceoleosa*. Sci. Hortic..

[45] Nakamura S., Sugimoto S., Matsuda H., Yoshikawa M. (2007). Medicinal flowers.: XVII.: New dammarane-type triterpene glycosides from flower buds of American ginseng, *Panax quinquefolium* L.. Chem. Pharm. Bull..

[46] Kim M.S., Jeon S.J., Youn S.J., Lee H., Park Y.J., Kim D.O., Kim B.Y., Kim W., Baik M.Y. (2019). Enhancement of Minor Ginsenosides Contents and Antioxidant Capacity of American and Canadian Ginsengs (*Panax quinquefolius*) by Puffing. Antioxidants.

[47] Shou W.U., Xiao G.U.O., Peng T.U., Yong J. (2022). Research progress on chemical constituents, biological activities, quality evaluation, and product development of *Panax quinquefolium*. China J. Chin. Mater. Medica.

[48] Yan N., Gai X., Xue L., Du Y., Shi J., Liu Y. (2020). Effects of *NtSPS1* Overexpression on Solanesol Content, Plant Growth, Photosynthesis, and Metabolome of *Nicotiana tabacum*. Plants.

[49] Zou H., Jiang H., Li L., Huang R. (2021). Integration of transcriptome and targeted metabolome profiling reveals hormone related genes involved in the growth of *Bletilla striata*. Sci. Rep..

[50] Ma D., Guo Y., Ali I., Lin J., Xu Y., Yang M. (2024). Accumulation characteristics of plant flavonoids and effects of cultivation measures on their biosynthesis: A review. Plant Physiol. Biochem..

[51] Ramaroson M.L., Koutouan C., Helesbeux J.J., Le Clerc V., Hamama L., Geoffriau E., Briard M. (2022). Role of Phenylpropanoids and Flavonoids in Plant Resistance to Pests and Diseases. Molecules.

[52] Khan Z., Midega C.A.O., Hooper A., Pickett J. (2016). Push-Pull: Chemical Ecology-Based Integrated Pest Management Technology. J. Chem. Ecol..

[53] Ma L., Ma S., Chen G., Lu X., Zhang C., Wei R., Feng X., Xu L., Zhang X., Chai Q. (2024). Transcriptomics and metabolomics revealed the synthesis and potential role of flavonoids in pea roots under continuous cropping obstacles. Physiol. Plant.

[54] Albert N.W., Thrimawithana A.H., McGhie T.K., Clayton W.A., Deroles S.C., Schwinn K.E., Bowman J.L., Jordan B.R., Davies K.M. (2018). Genetic analysis of the liverwort *Marchantia polymorpha* reveals that R2R3MYB activation of flavonoid production in response to abiotic stress is an ancient character in land plants. New Phytol..

[55] Li B., Fan R., Guo S., Wang P., Zhu X., Fan Y., Chen Y., He K., Kumar A., Shi J. (2019). The *Arabidopsis* MYB transcription factor, *MYB111* modulates salt responses by regulating flavonoid biosynthesis. Environ. Exp. Bot..

[56] Simiyu D.C., Bayaraa U., Jang J.H., Lee O.R. (2024). The R2R3-MYB transcription factor *PgTT2* from *Panax ginseng* interacts with the WD40-repeat protein *PgTTG1* during the regulation of proanthocyanidin biosynthesis and the response to salt stress. Plant Physiol. Biochem..

[57] Fang Y., Liu J., Zheng M., Zhu S., Pei T., Cui M., Chang L., Xiao H., Yang J., Martin C. (2023). *SbMYB3* transcription factor promotes root-specific flavone biosynthesis in *Scutellaria baicalensis*. Hortic. Res..

[58] Bai Y., Liu H., Pan J., Zhang S., Guo Y., Xian Y., Sun Z., Zhang Z. (2021). Transcriptomics and Metabolomics Changes Triggered by Inflorescence Removal in *Panax notoginseng* (Burk.). Front. Plant Sci..

[59] Yamashita M., Fujimori T., An S., Iguchi S., Takenaka Y., Kajiura H., Yoshizawa T., Matsumura H., Kobayashi M., Ono E. (2023). The apiosyltransferase celery *UGT94AX1* catalyzes the biosynthesis of the flavone glycoside apiin. Plant Physiol..

[60] Lu C., Wang H., Zheng D., Jia S., Xing Q., Wang Z., Li Q., Zhao L. (2024). Cloning and Direct Evolution of a Novel 7-*O*-Glycosyltransferase from *Cucurbita moschata* and Its Application in the Efficient Biocatalytic Synthesis of Luteolin-7-*O*-glucoside. J. Agric. Food Chem..

[61] Song C., Gu L., Liu J., Zhao S., Hong X., Schulenburg K., Schwab W. (2015). Characterization of *UGT71*, a Major Glycosyltransferase Family for triterpenoids, flavonoids and phytohormones-biosynthetic in plants. ResearchGate. Plant Cell Physiol..

[62] Okazawa A., Kusunose T., Ono E., Kim H.J., Satake H., Shimizu B., Mizutani M., Seki H., Muranaka T. (2014). Glucosyltransferase activity of *Arabidopsis UGT71C1* towards pinoresinol and lariciresinol. Plant Biotechnol..

[63] Hou M., Nie F., Zhao J., Ju Z., Yang L., Wang Q., Zhao S., Wang Z. (2023). New Glycosyltransferases in *Panax notoginseng* Perfect Main Ginsenosides Biosynthetic Pathways. J. Agric. Food Chem..

[64] Feng X., Bai S., Zhou L., Song Y., Jia S., Guo Q., Zhang C. (2024). Integrated Analysis of Transcriptome and Metabolome Provides Insights into Flavonoid Biosynthesis of Blueberry Leaves in Response to Drought Stress. Int. J. Mol. Sci..

[65] Song G., Yan Y., Guo C., Chen J., Wang Y., Wang Y., Zhang J., Gao C., Lian J., Piao X. (2024). Identification and Expression Analysis of R2R3-MYB Transcription Factors Associated with Flavonoid Biosynthesis in *Panax quinquefolius*. Int. J. Mol. Sci..

[66] Di P., Yan Y., Wang P., Yan M., Wang Y.-P., Huang L.Q. (2022). Integrative SMRT sequencing and ginsenoside profiling analysis provide insights into the biosynthesis of ginsenoside in *Panax quinquefolium*. Chin. J. Nat. Med..

[67] Wang Z.H., Wang X.F., Lu T., Li M.R., Jiang P., Zhao J., Liu S.T., Fu X.Q., Wendel J.F., Van de Peer Y. (2022). Reshuffling of the ancestral core-eudicot genome shaped chromatin topology and epigenetic modification in *Panax*. Nat. Commun..

[68] Pertea M., Kim D., Pertea G., Leek J.T., Salzberg S.L. (2016). Transcript-level expression analysis of RNA-seq experiments with *HISAT*, *StringTie*, and *Ballgown*. Nat. Protoc..

