# Supplementary material for: Integrated Transcriptomic and Metabolomic Analysis Reveals Tissue-Specific Flavonoid Biosynthesis and MYB-Mediated Regulation of UGT71A1 in Panax quinquefolius
Source: Int J Mol Sci. 2025 Mar 16;26(6):2669. doi: 10.3390/ijms26062669 (PMC11941809; doi:10.3390/ijms26062669)
Supplement: Supplementary file 1 [file ijms-26-02669-s001.zip › Supplementary figure.pdf]

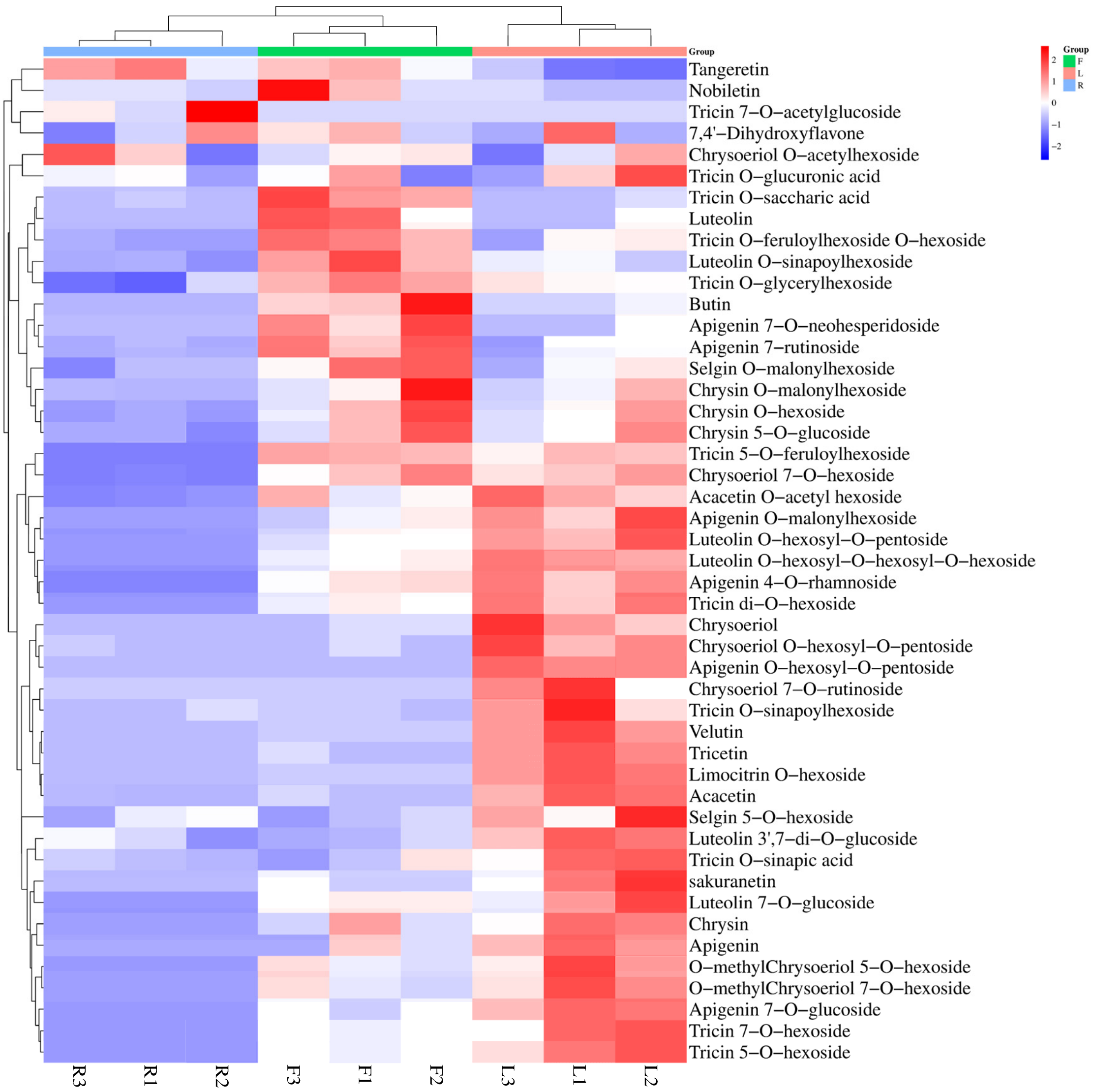

Figure S1 The Clustering Heatmap of Flavone from Different Tissues of *Panax quinquefolius.*

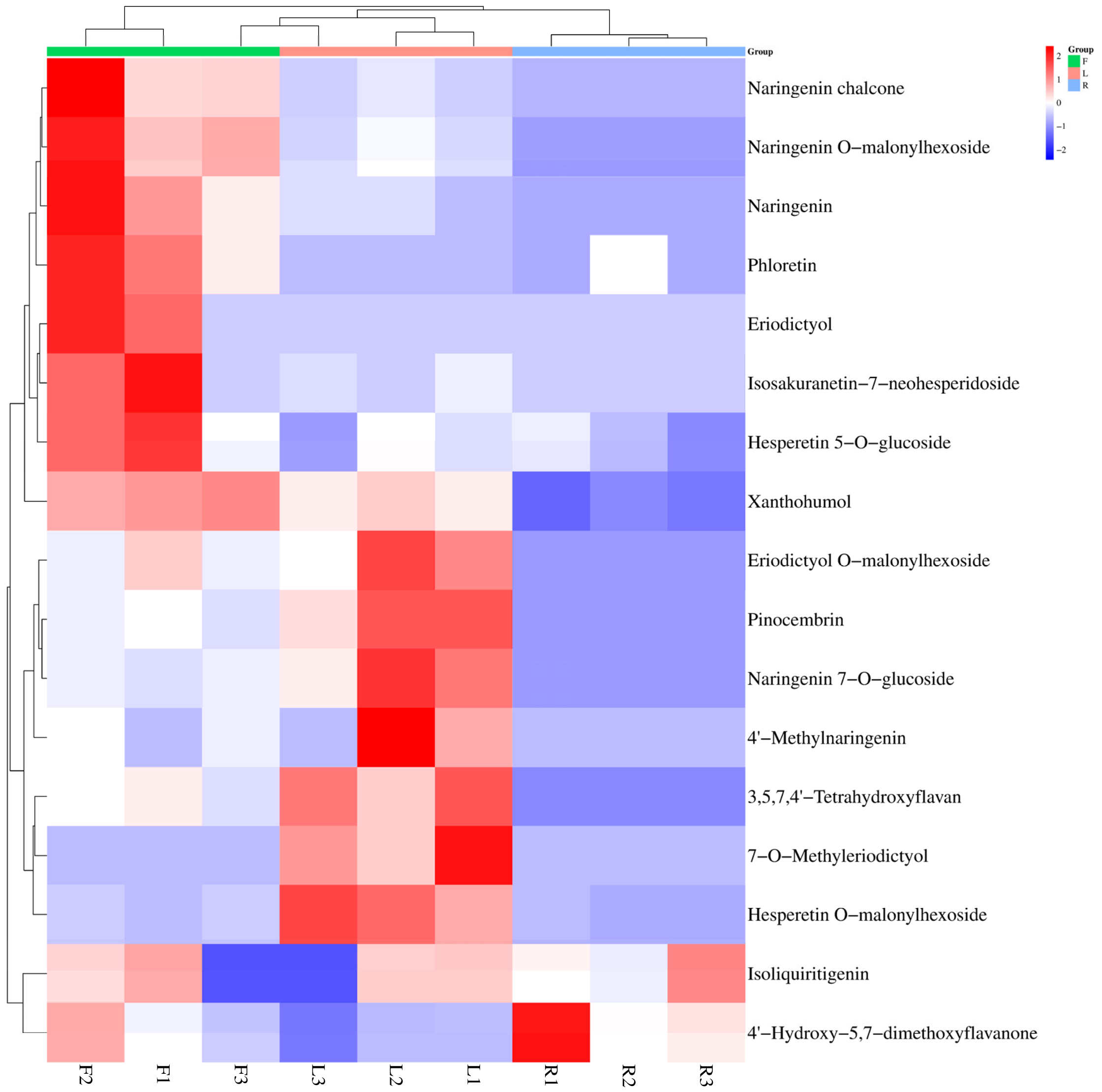

Figure S2 The Clustering Heatmap of Flavanone from Different Tissues of *Panax quinquefolius.*

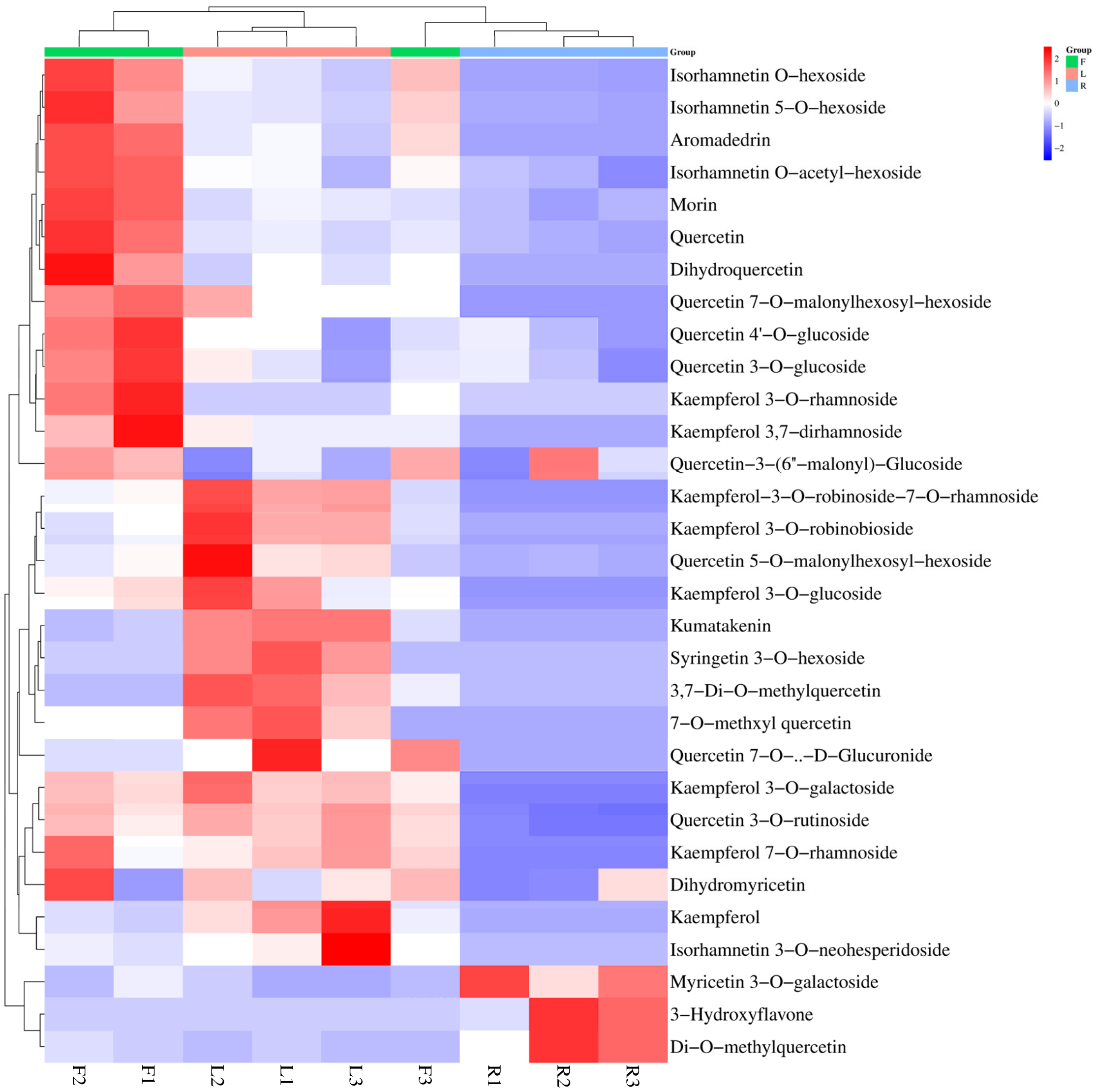

Figure S3 The Clustering Heatmap of Flavonol from Different Tissues of *Panax quinquefolius.*

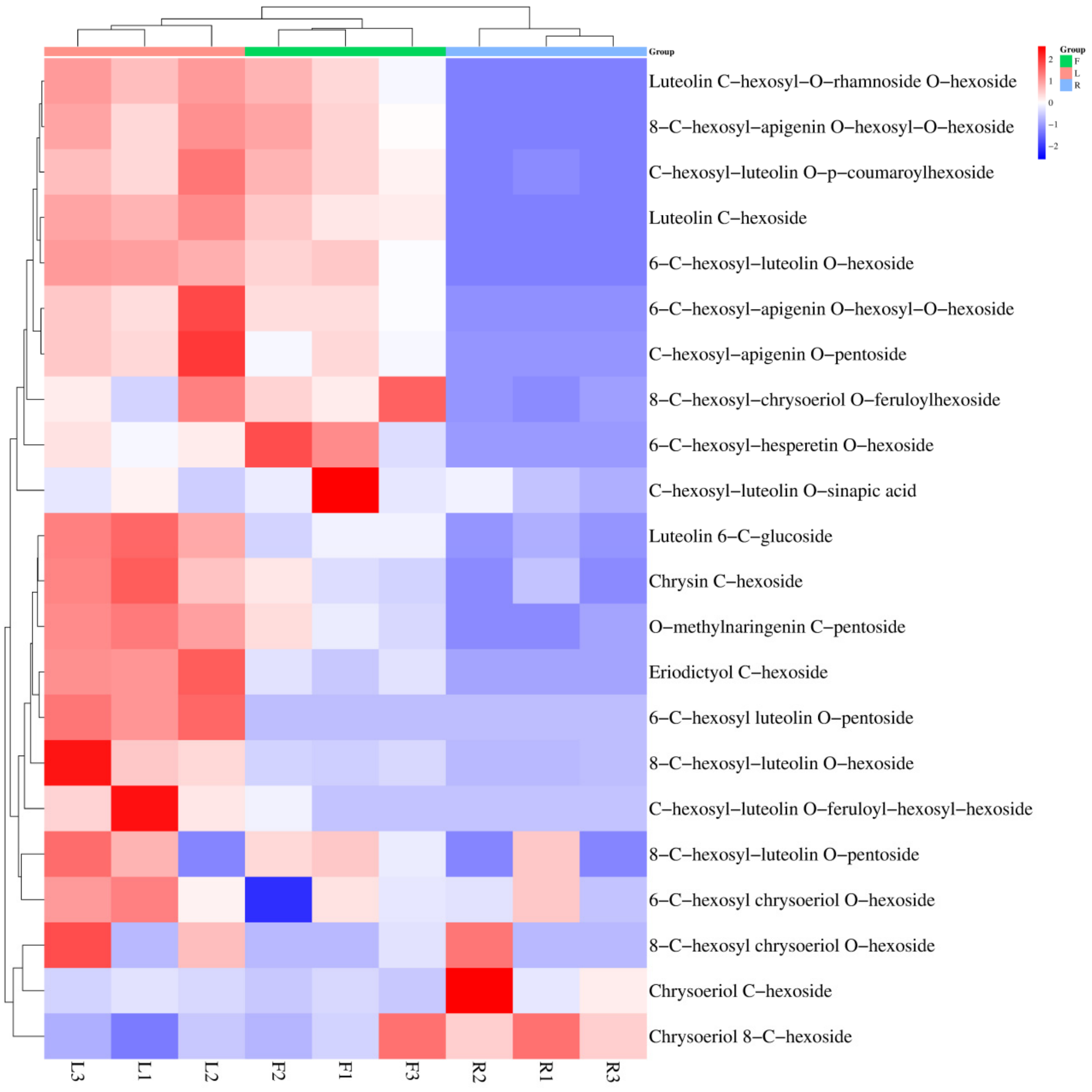

Figure S4 The Clustering Heatmap of Flavone C-glycosides from Different Tissues of *Panax quinquefolius*.

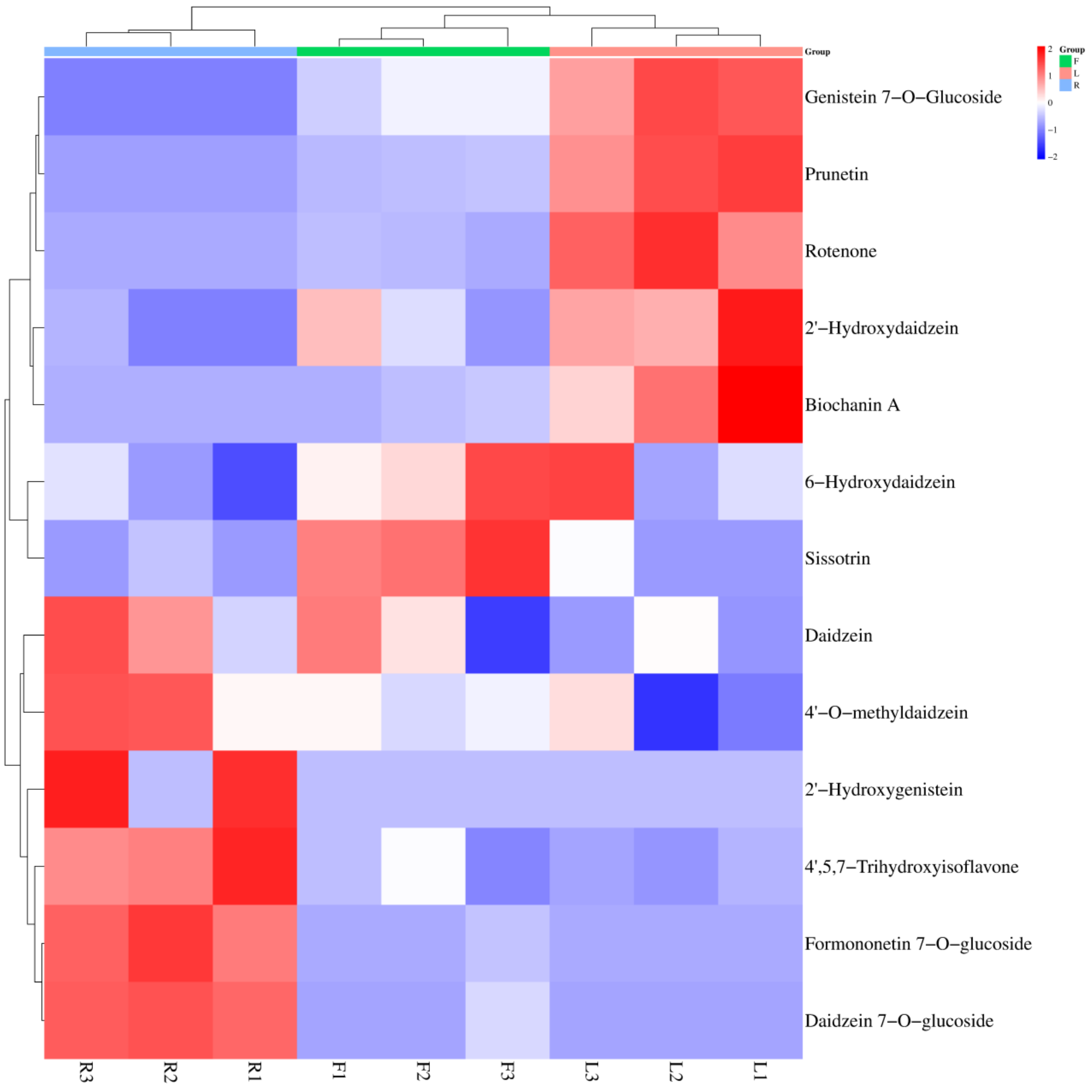

Figure S5 The Clustering Heatmap of Isoflavone from Different Tissues of *Panax quinquefolius.*

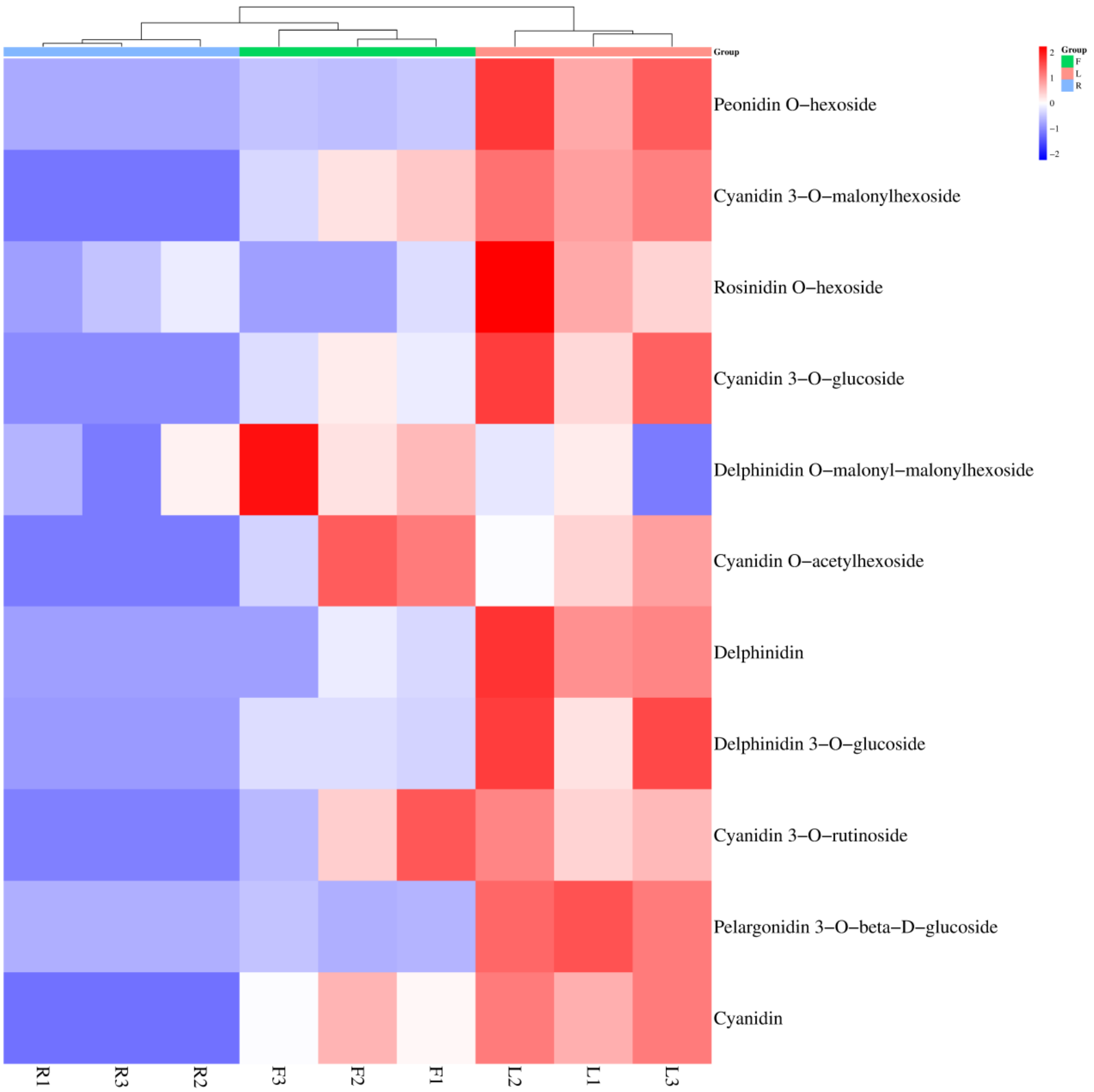

Figure S6 The Clustering Heatmap of Anthocyanins from Different Tissues of *Panax quinquefolius*.

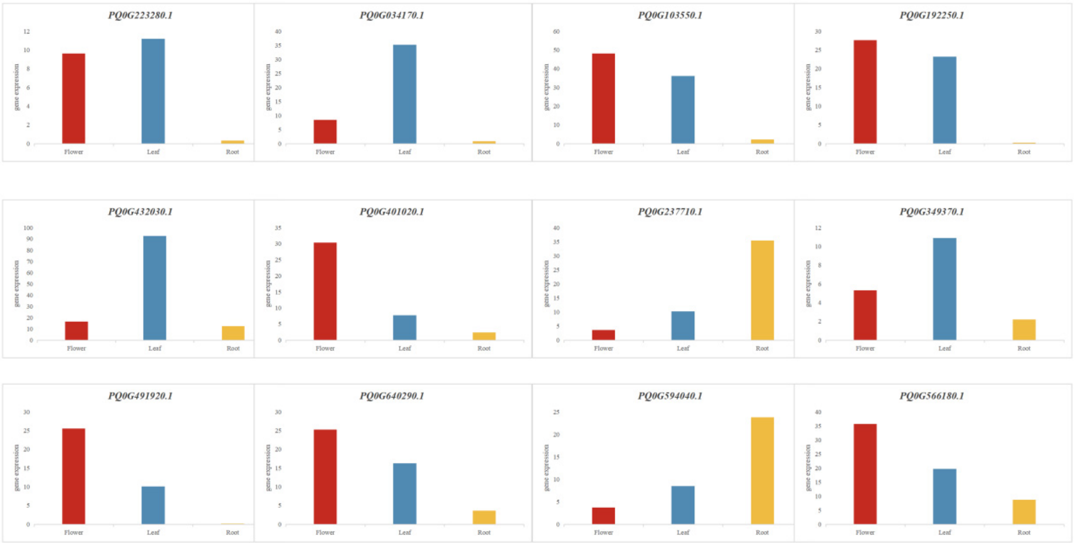

Figure S7 Gene Expression in *P. quinquefolius* RNA-seq Analysis.

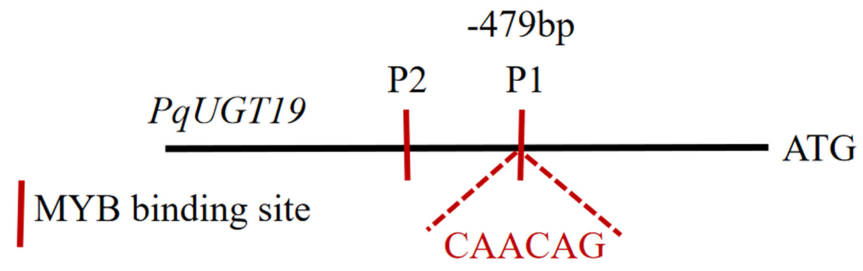

Figure S8 PqUGT19 promoter action element predicted by PlantCARE.

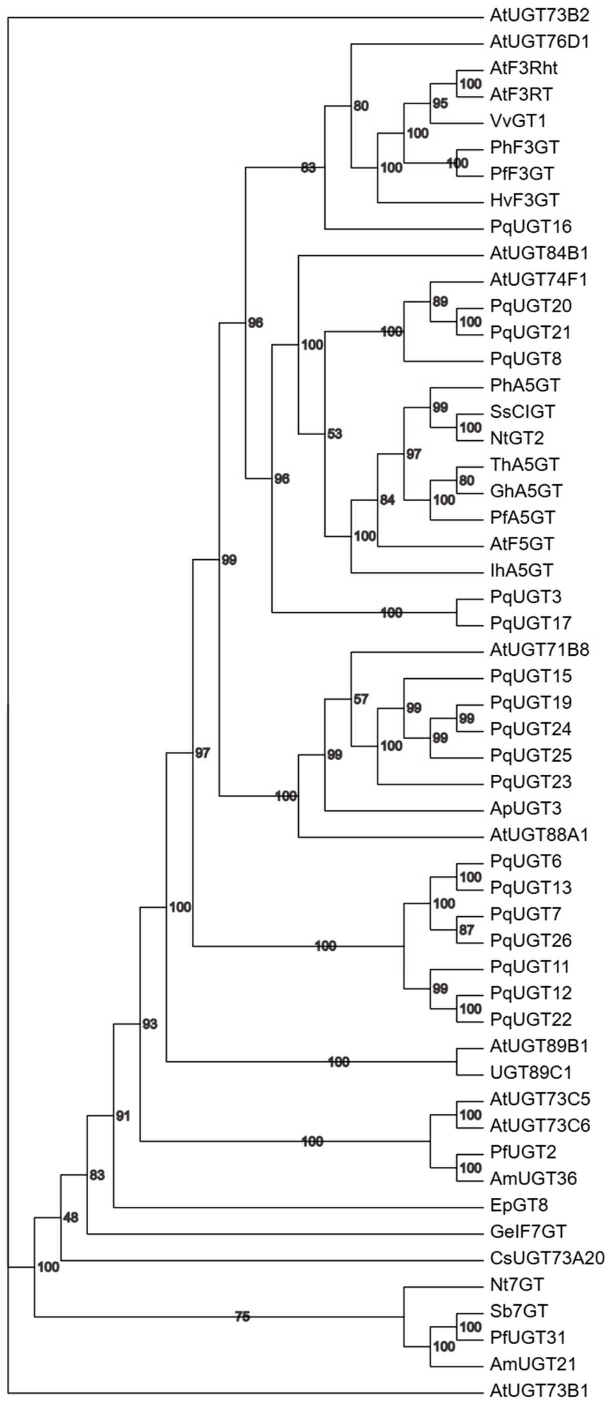

Figure S9 Phylogenetic tree of PqUGT and other reported UGTs.
